# Serum CGRP in migraine patients using erenumab as preventive treatment

**DOI:** 10.1186/s10194-022-01483-z

**Published:** 2022-09-12

**Authors:** Simone de Vries Lentsch, Ingrid M. Garrelds, A. H. Jan Danser, Gisela M. Terwindt, Antoinette MaassenVanDenBrink

**Affiliations:** 1 Department of Neurology, Leiden University Medical Centre, Leiden, The Netherlands; 2 Division of Vascular Medicine and Pharmacology, Department of Internal Medicine, Erasmus MC University Medical Center, Rotterdam, The Netherlands

**Keywords:** CGRP, Serum, Monoclonal antibodies, Migraine

## Abstract

**Aim:**

Serum levels of Calcitonin Gene-Related Peptide (CGRP)-like immunoreactivity (CGRP-LI) in migraine patients before and after starting treatment with erenumab were measured to evaluate the association with clinical treatment response.

**Methods:**

Blood samples were collected from the cubital fossa before (T0) and 2–4 weeks after (T1) starting treatment with erenumab. Clinical response was monitored using a daily headache e-diary. Serum levels of CGRP-LI, assessed using radioimmunoassay, were compared between T0 and T1, correcting for migraine reduction. In addition, for both T0 and T1, linear regression models were constructed using migraine reduction as outcome and serum CGRP-LI as independent variable, corrected for age, gender and monthly migraine days (MMD) at baseline.

**Results:**

Serum CGRP-LI did not differ between T0 and T1 (*p* = 0.30). However, there was an interaction between time and reduction in MMD (*p* = 0.01). Absolute reduction in MMD in the third month after treatment with erenumab was associated with serum CGRP-LI at T1, 2–4 weeks after starting treatment with erenumab (*p *= 0.003), but not with serum CGRP-LI at T0 (*p* = 0.24).

**Conclusion:**

Lower serum CGRP-LI 2–4 weeks after starting treatment with erenumab was associated with a higher reduction in migraine days after three months of treatment. Although the underlying mechanisms remain to be determined, this suggests that changes in CGRP levels, shortly after starting erenumab, are important for its clinical effect.

## Highlights

• Lower serum levels of calcitonin gene-related peptide (CGRP)-like immunoreactivity (CGRP-LI) in migraine patients at 2–4 weeks after starting treatment with erenumab were associated with better treatment response after three months.

• Early changes in serum CGRP may be important for the clinical effect of erenumab in migraine.

## Introduction

Activation of the trigeminovascular system and the subsequent release of calcitonin gene-related peptide (CGRP) play an important role in the pathophysiology of migraine [1]. CGRP levels have been shown to be elevated in the jugular vein during spontaneous migraine attacks [2], while in chronic migraine patients the interictal CGRP levels were also found to be elevated [3]. In addition, infusion of CGRP in migraine patients induces a migraine-like headache, similar to the subject’s spontaneous attack, in approximately 60% of patients [4].

The development of monoclonal antibodies directed against CGRP (eptinezumab, fremanezumab and galcanezumab) or its receptor (erenumab) has been a major advancement in the treatment of migraine. Unfortunately, not all migraine patients can be considered responders to this type of medication. In clinical trials, approximately 50% of migraine patients had 50% reduction in monthly migraine days (MMD) in the last month of treatment or as mean response over several months of treatment. In patients with previous failure to 2–4 prophylactics 30–40% achieved a 50% reduction, with, as expected, a lower placebo response [5–10]. A real-life study in our center in those patients with ≥ 8 MMD and failure on 2–4 prophylactics showed that of all patients 60% had ≥ 30% MMD reduction in at least half of their treatment period (≥ 3/6 months) [11].

Increasing the understanding of the pathophysiological effects of anti-CGRP (receptor) antibodies and uncovering differences between responders and non-responders to this treatment will help to improve migraine care even further. While it has been suggested that serum CGRP decreases when migraine attack frequency decreases [3], another small study suggested an increase in serum CGRP levels after long term blockade of the CGRP receptor with erenumab [12], but no clear underlying mechanisms were proposed. Indeed, a lot is still unknown about the clearance of CGRP, which may be caused by endopeptidases, but in addition possibly also by neuronal reuptake [13].

In the present study, we assessed serum CGRP levels in migraine patients before and 2–4 weeks after starting treatment with erenumab and evaluated the association with the clinical treatment response.

## Methods

### Participants

All patients that started treatment with erenumab in the Leiden Headache Center, a national referral centre, were invited to participate. They were all diagnosed with migraine, episodic or chronic, with or without aura, by a neurology resident in consultation with a neurologist with headache expertise or by a neurologist with a headache expertise, according to the ICHD-3 criteria [14]. None of the patients had a second primary headache disorder. Only tension type headache was allowed, as this is common in patients with chronic migraine [14]. Given the restricted availability of erenumab, all patients had at least 8 migraine days per month, and failed on at least 4 migraine prophylactics (meaning being ineffective, discontinued because of side effects or being contraindicated), including at least a betablocker, candesartan, valproate and topiramate. None of the patients had medication overuse headache.

Approval for this study was obtained from the LUMC Medical Ethical Committee and all participants gave written informed consent.

### Treatment

Patients were treated with erenumab 70 mg, administered subcutaneously once every four weeks. No additional prophylactic treatment was used.

### Headache diary

The clinical response to erenumab was monitored using a validated daily headache e-diary [11, 15, 16]. This diary contains questions on the presence of headache, headache characteristics, accompanying symptoms and the use of acute migraine medication. In case of a headache, an automated algorithm based on the ICHD-3 criteria determined whether it was a migraine day. Additionally, days on which a triptan was taken, as well as aura without headache symptoms, were also counted as migraine days. Patients started this diary at least 4 weeks before starting treatment (the baseline period). In line with clinical trials [7], the clinical response was assessed by comparing MMD in week 9–12 (i.e. after three doses of erenumab) to that in the 4 week pre-treatment baseline observation period. A month is defined as 28 days (4 weeks).

#### Serum CGRP assays

Patients were invited to the hospital before starting treatment with erenumab (T0) and 2–4 weeks (after T_max_, but before the second dosing) after starting treatment with erenumab (T1). At both time points blood samples were collected from the antecubital vein, while subjects rested in a sitting position. The blood was then allowed to clot and was centrifuged at room temperature for 20 min at 622 *g*/2000 rpm to separate serum. Samples were then immediately stored at -80 °C in aliquots of 500 µL until analyzed.

For radioimmunoassay (RIA), a commercial kit (*CGRP (Human) - RIA Kit* (Phoenix pharmaceuticals, Burlingame, California, United States), detection range 0.53–660 pmol/l), was used following manufacturers’ instructions to measure CGRP-like immunoreactivity (CGRP-LI) levels. Biochemical assays were performed by an experienced lab technician who was blinded to the patient identity, study day and treatment effect of erenumab. All samples were analyzed in the same laboratory, under the same environmental conditions, and using the same batch for samples from different patients and different study days, to avoid a possible batch effect. Samples with values outside the detection range were set on the limits of the detection range.

#### Statistics

Sample size was based on the available data. Baseline characteristics, including, sex, age, headache diagnosis and baseline headache measures were summarized using means and standard deviations or frequencies and proportions. For each patient the clinical response to erenumab was determined by calculating the absolute reduction in migraine days in the third month (week 9–12) after initiating treatment compared to the baseline month (4 weeks before starting treatment).

As serum CGRP-LI levels were highly skewed, a log transformation was applied, and these log-transformed values of CGRP-LI levels were used in all statistical analyses. However, for the sake of clarity, in the result section CGRP-LI levels are presented, without log transformation, as medians with interquartile ranges. To relate our CGRP measurements to measurements performed earlier by others, CGRP-LI levels at T0 were related to age and sex, with a Pearson correlation and an independent t-test, respectively. Comparisons between T0 and T1 were made using a repeated measurements model, with absolute reduction in monthly migraine days added as a covariate to assess the relation between change in serum CGRP-LI and change in migraine frequency.

To investigate the predictive value of serum CGRP-LI for the clinical response, two linear regression models were made with absolute migraine reduction as the outcome variable and with sex, age, migraine days at baseline as covariates. In our primary analysis, log serum CGRP-LI at T0, and in our secondary analysis log serum CGRP-LI at T1, was added as an independent variable.

In all analyses a two-sided *p*-value < 0.05 was considered to indicate significant differences. All statistical analyses were performed using IBM SPSS Statistics for Windows, version 25 (IBM Corp., Armonk, N.Y., USA).

## Results

In total, 96 participants started treatment with erenumab. Two patients discontinued treatment before the three month follow-up period ended, and thus were excluded from all analyses. CGRP measurements of 5 patients were missing at follow-up, since one patient was not able to attend the second visit because of a debilitating migraine attack, and four measurements were missing because of COVID-19 measures, when patients were not allowed to come to the hospital for nonurgent (research) issues. These patients were excluded regarding analyses with follow-up measurements. In total, 94 patients were included, of which 79 were women. Baseline characteristics are described in Table 1. At T0, three values were below and one above the detection range. At T1, six values were below and one above the detection range.

**Table 1:** Baseline characteristics (*n* = 94)

Characteristic	
Women, n (%)	79 (84)
Age, mean ± SD (years)	42 ± 12.6
Migraine without aura, n (%)	60 (64)
Episodic migraine, n (%)	52 (55)
MMD baseline, mean ± SD	13.7 ± 5.7
MHD baseline, mean ± SD	16.5 ± 6.1
Failed prophylactics, mean ± SD	5.0 ± 1.0

*MMD* monthly migraine days, *MHD* monthly headache days, A month is defined as 28 days. Baseline = 28 days before starting treatment

### Baseline comparisons

Serum CGRP-LI at T0 was not significantly different between women (median (IQR) CGRP-LI = 15.1 (8.3–47.8) pmol/l) and men (median (IQR) CGRP-LI = 10.6 (6.3–29.7) pmol/l) (*p* = 0.12). Serum CGRP-LI at T0 levels were negatively correlated to age (r = -0.26, *p* = 0.01).

#### Erenumab

**Fig. 1:**
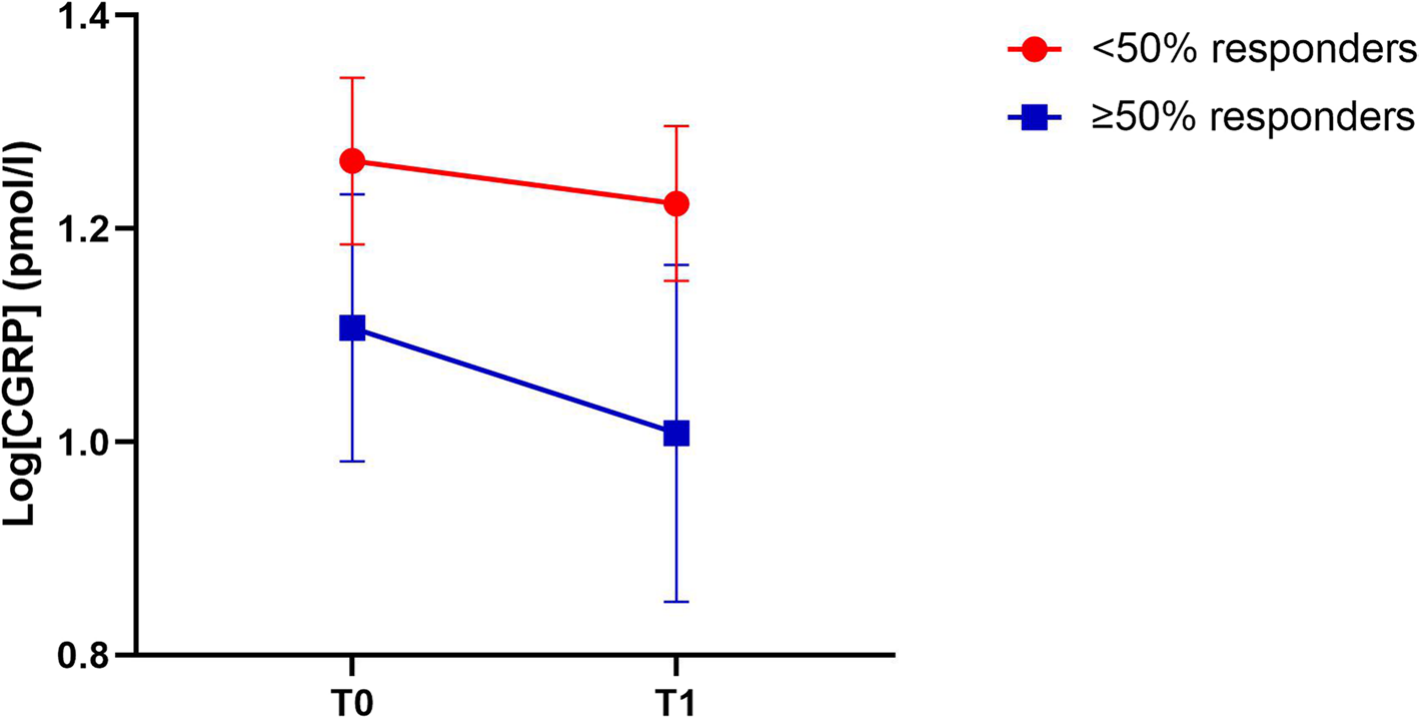
Change in serum log[CGRP-LI] between T0 and T1 (2–4 weeks after starting erenumab) separated for patients with < 50% and ≥ 50% reduction in monthly migraine day (MMD) reduction after three months of treatment. Data presented as mean ± SEM

Serum CGRP-LI did not differ between T0, before starting erenumab, (median (IQR) CGRP-LI: 14.1 (8.2–33.9) pmol/l) and T1, after 2–4 weeks treatment (median (IQR) CGRP-LI: 13.8 (7.0–33.1) pmol/l) (F(1, 86) = 1.1, *p* = 0.30). However, there was an interaction between time and reduction in MMD (F(1, 86) = 6.8, *p* = 0.01). To visualize the interaction between migraine reduction and change in serum CGRP-LI, we present a line graph, separated for < 50% and ≥ 50% responders (Fig. 1).

Tables 2 and 3 present the β-coefficients and p-values of the linear regression analyses with serum CGRP-LI at T0 and serum CGRP-LI at T1 as predictor for the clinical response. Absolute MMD reduction after three months of treatment with erenumab was associated with serum CGRP-LI at T1 (β = -2.13, *p* = 0.003), but not with serum CGRP-LI at T0 (β = -0.80, *p* = 0.24).

**Table 2:** Linear regression analysis with log-transformed serum CGRP-LI levels (mol/l) T0

Variable	β (95% CI)^1^	p	β (95% CI)^2^	p
Age	0.07 (-0.001–0.13)	0.05	0.07 (0.004–0.14)	**0.04**
Sex	1.92 (-0.41–4.25)	0.10	2.50 (0.17–4.82)	**0.04**
Migraine days baseline	0.09 (-0.06–0.24)	0.24	0.11 (-0.04–0.26)	0.14
Serum CGRP-LI	-1.033 (-2.37–0.30)	0.13	-0.80 (-2.16–0.55)	0.24
*N* = 94. ^1^Simple linear regression. ^2^multiple regression, corrected for all tested variables. CI = confidence interval. T0 = baseline, before starting treatment with erenumab. The outcome is absolute reduction migraine days during month 3 after starting treatment with erenumab compared to baseline. One month is defined as 28 days.				

**Table 3:** Linear regression analysis with log-transformed serum CGRP-LI levels (mol/l) T1

Variable	β (95% CI)^1^	p	β (95% CI)^2^	p
Age	0.07 (-0.001–0.13)	0.05	0.044 (-0.03 − 0.12)	0.24
Sex	1.92 (-0.41–4.25)	0.10	2.979 (0.65–5.31)	**0.01**
Migraine days baseline	0.09 (-0.06–0.24)	0.24	0.10 (-0.06–0.25)	0.22
Serum CGRP-LI	-2.12 (-3.44 - -0.80)	**0.002**	-2.13 (-3.52 - -0.73)	**0**.**003**
N = 89. ^1^Simple linear regression. ^2^multiple regression, corrected for all tested variables. CI = confidence interval. T1 = 2–4 weeks after starting treatment with erenumab. The outcome is absolute reduction migraine days during month 3 after starting treatment with erenumab compared to baseline. One month is defined as 28 days.				

## Discussion

Lower serum CGRP-LI levels measured 2–4 weeks after starting treatment with erenumab are associated with a higher migraine reduction after three months. Serum CGRP-LI levels before start of treatment with erenumab were not associated with clinical response.

Previous small studies suggested that chronic migraine patients may have higher serum CGRP levels than episodic migraine patients [2, 3]. It was also suggested that serum CGRP levels in episodic migraine patients with a history of chronification are within the range of episodic migraine CGRP levels [3]. This may suggest that, when migraine attack frequency decreases, spontaneously or due to successful treatment, one might expect to measure lower serum CGRP levels. In contrast, a small proof-of-concept study, in which CGRP levels were measured before, after one month and after six months of treatment with erenumab, suggested an increase [12]. While the association with the clinical response was not described and the sample size was too small (*n* = 7) to demonstrate statistical significance, it was suggested that serum levels of CGRP did not change in the first month, but tended to increase after six months [12].

The present study focused on identifying a possible early predictor for clinical response to treatment with erenumab. We deliberately chose to measure CGRP-LI early (after 2–4 weeks), as we did in another recent study from our group [17], so we could analyze the association with the clinical response, and at the same time rule out whether changes in CGRP-LI were a secondary effect due to a change in migraine days. Although CGRP-LI levels were not different between T0 and T1, an interaction was found with migraine reduction after three months. In addition, lower serum CGRP-LI levels 2–4 weeks after the first erenumab injection were associated with a larger monthly migraine day reduction after three months, while CGRP-LI levels at T0 were not associated with the clinical response. Moreover, the CGRP-LI levels at T1 were not associated with migraine reduction in the first two months (results not shown). These findings combined suggest that, promptly after starting anti-CGRP treatment, there are relevant changes in serum CGRP-LI that are important for the clinical effect and these changes are not a secondary effect of a decrease in migraine frequency. Interestingly, although the clinical effect of erenumab is already evident in the first month, the monthly migraine days seem to decrease further after the first month, which seems to be in line with what could be expected given the long half-life of the mAbs [18, 19].

Much is still unknown about the effects of blocking the CGRP receptor. Indeed, it does not seem unlikely that serum levels of CGRP would increase due to upregulation after long term blockade of the CGRP receptor [12, 20]. However, interactions between CGRP activity and several other peptides (and/or their receptors) probably induce a more complex cascade of events, that could either increase or decrease serum CGRP. CGRP can act through both the CGRP and the amylin 1 receptors, with unknown effects on further CGRP release [21]. In addition, CGRP release may be indirectly influenced by changing activity of the sympathetic nervous system and endogenous endothelin-1 release, which may modulate CGRP release through the TRPV1 receptor [22, 23]. Lastly, CGRP might regulate its own release through presynaptic mechanisms [24].

A strong feature of our study is the use of a daily e-diary. The time-lock reduces the risk of recall bias, and with the automated algorithm (reduction in) migraine days could be determined accurately. In addition, while CGRP has a short half-life and is rapidly cleared from the blood, all our blood samples were collected under the same circumstances and processed and stored directly after blood draw. The association between CGRP-LI levels and age and sex have been described in the literature before[13, 25]. Although we could only demonstrate a numerical and not statistical difference between men and women (most likely due to a lack of statistical power in our population including only a limited number of men), we did see an association between CGRP-LI levels and age in our samples, supporting the validity of the CGRP assessment [13].

Previous studies demonstrated that CGRP-LI levels measured in the antecubital vein are generally lower than in the jugular vein, and differences between migraine patients and controls are generally smaller in antecubital vein than in jugular vein samples. In our study, this same phenomenon might have caused insufficient power for our comparison between T0 and T1. However, we decided to use the antecubital vein for blood sampling because it is more patient friendly, and because previous studies demonstrated that the antecubital vein is suitable to measure CGRP-LI in migraine patients. Moreover, a lot is still unclear about CGRP-LI measurements in human serum, where CGRP most probably has been degraded into smaller fragments by endogenous peptidases [26]. Therefore, we consider data on CGRP-LI serum levels important within a study, where all samples were treated identically as described above, but we remain cautious about an interpretation of the absolute levels that we measured. A second limitation is that, due to the high migraine frequency in our study population, blood sample collection did not always take place on an interictal day. However, there was no difference between the CGRP-LI levels on migraine days and non-migraine days (data not shown). This is probably due to the fact that all our patients had high frequent episodic or chronic migraine, in whom interictal CGRP-LI levels are most likely already increased [2, 3]. Thirdly, the significant effect of sex on the clinical response needs to be interpreted with caution as there were very few men in our analysis and our study was not powered to determine a difference in effectiveness of monoclonal CGRP-antibodies between men and women. This needs to be investigated in a separate study [27].

Currently, in many countries treatment with anti-CGRP (receptor) antibodies is only available to a subset of patients, namely patients with a high monthly attack frequency and/or who already demonstrated not to respond to multiple preventive treatments. Data from clinical trials and real life data show that not all migraine patients have a successful migraine reduction in response to treatment with anti-CGRP (receptor) antibodies [11 28]. Even though anti-CGRP (receptor) antibodies were specifically developed for the preventive treatment of migraine, it is yet unclear why some patients do not respond and others are responders. Recently, we demonstrated that CGRP-mediated trigeminovascular activity before initiating erenumab partly may explain this clinical response [17]. However, it is of utmost importance to increase the understanding of response to anti-CGRP treatment even further and to uncover reasons for (non-)response. Future studies, in larger patient cohorts, may need to be performed to confirm our results. In addition, future research needs to unravel the exact mechanisms behind the relation between serum CGRP levels and clinical response to erenumab. Finally, measuring CGRP in patients receiving an anti-CGRP antibody might provide additional information on the expectations of effects of this treatments.

## Conclusion

Lower serum levels of CGRP-LI shortly after starting treatment with erenumab were associated with a higher reduction in migraine days after three months of treatment. While the underlying mechanisms remain to be determined, this suggests that early changes in CGRP-LI levels shortly after starting erenumab, are important for its clinical effect.

## Data Availability

Data not published within the article is available from the corresponding author on reasonable request.

## Abbreviations

MMD: Monthly migraine days
MHD: Monthly headache days
CGRP: Calcitonin gene-related peptide
ICHD: International Classification of Headache Disorders
RIA: Radioimmunoassay
TRPV1: Transient receptor potential cation channel subfamily V member 1
